# *Daphnia magna* trade‐off safety from UV radiation for food

**DOI:** 10.1002/ece3.8399

**Published:** 2021-11-25

**Authors:** Marcus Lee, Lars‐Anders Hansson

**Affiliations:** ^1^ Department of Biology Aquatic Ecology Lund University Lund Sweden

**Keywords:** 3D tracking, behavior, foraging, UV threat, vertical position, zooplankton

## Abstract

Research on diel vertical migration (DVM) is generally conducted at the population level, whereas few studies have focused on how individual animals behaviorally respond to threats when also having access to foraging opportunities. We utilized a 3D tracking platform to record the swimming behavior of *Daphnia magna* exposed to ultraviolet radiation (UVR) in the presence or absence of a food patch. We analyzed the vertical position of individuals before and during UVR exposure and found that the presence of food reduced the average swimming depth during both sections of the trial. Since UVR is a strong driver of zooplankton behavior, our results highlight that biotic factors, such as food patches, have profound effects on both the amplitude and the frequency of avoidance behavior. In a broader context, the trade‐off between threats and food adds to our understanding of the strength and variance of behavioral responses to threats, including DVM.

## Graphical abstract

Research on individual behavior in zooplankton, a globally important group of organisms, is lacking. We used 3D tracking of individual water fleas to test how the response to threats is modified by foraging opportunities. In the presence of food, threat response is reduced.

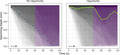

## 1 INTRODUCTION

Energy may be considered the currency with which all life is concerned. However, the acquisition of energy is far from simple in the complex landscapes that all organisms live. Foraging is a risky activity as during the search for food, individuals are more often exposed to threats. Therefore, foraging should occur only until the benefits are equal to the costs of further foraging (Brown & Kotler, 2004). The decisions whether to forage or avoid a threat, for example, predation, have been both theoretically (Brown & Kotler, 2004) and empirically studied in countless taxa, such as invertebrates (Kohler & McPeek, 1989), amphibians (Eklöv & Halvarsson, 2000), birds (Olsson et al., 2002), and mammals (Brown & Kotler, 2004).

One of the largest foraging movements, in terms of biomass, is diel vertical migration of zooplankton (DVM), which is unique to aquatic environments (Hays, 2003; Lampert, 1989). This is the large scale and daily movement from surface waters during the night to the deep waters during sunlight hours, occurring in both marine and freshwater systems. Both the proximate and ultimate mechanisms of this movement have been investigated for decades with a prominent explanation being food‐rich surface waters serving as an opportunity, coupled with increased predation risk from visually hunting predators acting as a threat (Bandara et al., 2021; Lampert, 1989). However, DVM has been documented from environments where such predation was not a strong predictor, suggesting that other factors are important in the cost–benefit analysis when foraging (Muluk & Beklioglu, 2005). Under these scenarios, solar UV radiation (UVR), which is the high‐energy wavelengths between 280–400 nm (Tucker & Williamson, 2011), is often considered an important environmental driver of this foraging migration.

UVR is an appreciable risk in aquatic environments (Hansson & Hylander, 2009), with considerable negative effects being reported at many trophic levels (Peng et al., 2017). With regard to zooplankton, UVR has been shown to reduce survival (Rautio & Korhola, 2002), impede growth and decrease fecundity (Grad et al., 2001), as well as inducing behavioral avoidances (Hansson et al., 2016; Heuschele et al., 2017). Despite the plethora of negative effects, UVR is attenuated over depth providing a refuge deeper in the water column, and therefore, penetration of UVR is highly variable between lakes, penetrating between 0.5 m and +20 m varying with the lake transparency (Tucker & Williamson, 2011). Particularly, clear lake communities, such as alpine lakes, are far more susceptible to the effects of UVR than communities in lakes high in colored dissolved organic matter, which has been demonstrated to affect behavior of zooplankton (Wolf & Heuschele, 2018). Along with variation between lakes, the rapid changes in cloud cover create acute temporal variation that demands equally instant changes in response to UVR fluctuations (Hansson et al., 2016).

Certain concepts such as “ideal‐free distribution” which consider resource availability have been very successful in explaining the vertical distribution of zooplankton under predation risk and other abiotic variables (Maszczyk et al., 2018, 2021). However, UVR has not specifically been investigated in such models, in spite of the confirmation of UVR being an important driver in the spatial positioning of zooplankton through field observations or population‐level experiments (Leech & Williamson, 2001; Rose et al., 2012). Therefore, the objective of this study was to investigate the mechanistic role of spatial heterogeneity in determining foraging decisions of a common zooplankter under risk conditions. Here, we specifically focus on how individual *Daphnia magna* respond to the trade‐off between remaining in the food‐rich surface water and avoiding the potentially harmful UV radiation. We hypothesize that *D*. *magna* will have weaker response to UVR threats when in the presence of foraging opportunities, that is, that they trade‐off safety for food.

## 2 METHODOLOGY

### 2.1 Culture conditions

*Daphnia magna* were obtained from a laboratory population initially inoculated with several genotypes originating from Lake Bysjön (55.6753 lat, 13.5452 long). This culture was maintained in a 12‐L plastic aquarium at 20°C with a 16:8‐h light:dark photoperiod for over 100 generations. They were fed *ad libitum* with a predominantly *Tetradesmus obliquus* algal suspension. Three days prior to the behavioral assay, individuals were randomly selected and transferred to pre‐experimental holding jars to reduce the effects of competition. These consisted of a 100‐ml jar filled with 80 ml aged tap water and individuals were fed with 120,000 cells ml^−1^ of a single species culture of *T*. *obliquus* (NIVA‐CHL 6) for two days before being transferred to a new jar with the same conditions for one further day. This ensured a high quality and quantity of food was available, especially immediately prior to the behavioral assay.

### 2.2 Behavioral assay

Each *Daphnia* was individually assayed by adapting a proven protocol for tracking the swimming behaviors of mm‐sized zooplankton (Ekvall et al., 2013; Langer et al., 2019). Individuals were transferred to 2‐ml centrifuge tubes and labeled with fluorescent nanoparticles (655 ITK Carboxyl quantum dot, fluorescent at 655 nm; Life Technologies, Carlsbad, California, USA, Prod. Nr.: Q21321MP). Assays were conducted in an experimental aquarium (0.2 × 0.2 × 0.75 m), upon which sits a lighting unit with 8 blue light‐emitting diode (LED) arrays, acting as excitation lights for the fluorescent nanoparticles. In the center of the lighting array, there is a UVR LED (100 µW/cm^2^; ENFIS UNO Tag Array Ultra‐Violet 375 nm UV‐A ENFIS LIMITED, Swansea, United Kingdom) which simulates UVR threat. Facing the aquaria are four synchronized digital cameras (Pike F‐210C, Allied Vision Technologies GmbH, Stadtroda, Germany), which allows the recording of videos and the triangulation of 3D coordinates. Further details on the labeling process and experimental system can be found in Ekvall et al. (2013) and Langer et al. (2019).

To test swimming behaviors in the presence of a high‐density food patch, *Daphnia* were either tested in the aquarium with 200 ml of *T*. *obliquus* culture, which was heated to 42°C and slowly introduced to the surface of the water, or 200 ml of 24–48 h aged tap water heated to the same extent and introduced in the same manner. The temperature difference between the aquarium water and the introduced medium created a spatially explicit food patch (*n* = 46) or “control” temperature patch (*n* = 28) at the surface. We performed this experiment in blocks; that is, we used up to a maximum of 5 separate individuals sequentially in the same arena before removing all the water, cleaning the recording arena and resetting the conditions. We measured the algal concentration, temperature, and ultraviolet irradiance in situ. The algal concentration was measured at 3 depths (surface, middle, and bottom; 0, 25 & 75 cm, respectively) using the AlgaeLabAnalyzer (bbe Moldaenke GmbH, Schwentinental, Germany) at the end of each experimental block for a conservative estimate of the spatial variation in the food patch. Temperature was measured at the beginning of each block as to ensure the starting temperature was within the thermal tolerance for *Daphnia magna* (5–30°C) (Seefeldt & Ebert, 2019). Repeated measurement of UV irradiance rapidly disperses the food patch throughout the water column; therefore, this was only measured once by lowering a radiometer (IL 1400A; International Light; Newburyport, MA, USA) with sensors for UV‐A (320–400 nm) through the water column, before the data collection began.

*Daphnia* have been suggested to sense food patches using visual, mechanical, and olfactory cues; however, there are conflicting reports (van Gool & Ringelberg, 1998; Roozen & Lurling, 2001). To ensure individuals had the same opportunity to detect the food patch, we carefully introduced each individual to the surface water of the aquarium, directly exposing them to the patch, and the assay began immediately. The behavioral recording lasted for two minutes with two distinct phases. The first minute may be considered the acclimation phase, and the second was the threat phase, mimicking solar radiation, whereby the UVR LED was turned on. Due to the location of the food patch at the top of the aquarium, the positioning of the UVR LED creates a direct trade‐off between foraging opportunities and threat. This threat has been repeatedly shown to elicit a response in *Daphnia magna* while in clear water (Ekvall et al., 2020; Hansson et al., 2016; Heuschele et al., 2017). Using MATLAB, we obtained coordinates from the tracks of the videos, and using R v 3.6.2 (R Core Team, 2019), we were then able to extract three‐dimensional positions from the list of *XYZ* coordinates. Missing coordinates between two known coordinates were interpolated using the package *zoo* (Zeileis & Grothendieck, 2005).

### 2.3 Data handling and analysis

All data handling and statistical analyses were conducted with the software R v 3.6.2. The data and code for this study are archived online (Lee & Hansson, 2021). For all analyses, we used the median of each individual recorded as the “average” to limit the influence of extreme values, unless otherwise specified. Since we obtained the positions during both the acclimation and UVR phase, we subsequently utilized the average vertical position in either phase as the dependent variable. We then analyzed the average depth using Mann–Whitney *U* tests due to non‐normality. We also utilized linear regression to investigate correlations between the mean swimming depth during each recording block and the environmental conditions during that behavioral assay. If UVR level alone would explain the depth distribution in the water column, we would expect a similar number of animals above and below the same UV level between both treatments. This was tested by comparing the frequency of animals at the UVR level corresponding to the depth of mean chlorophyll‐a both with and without a food patch using Pearson's *chi*‐*squared*.

## 3 RESULTS

**FIGURE 1:**
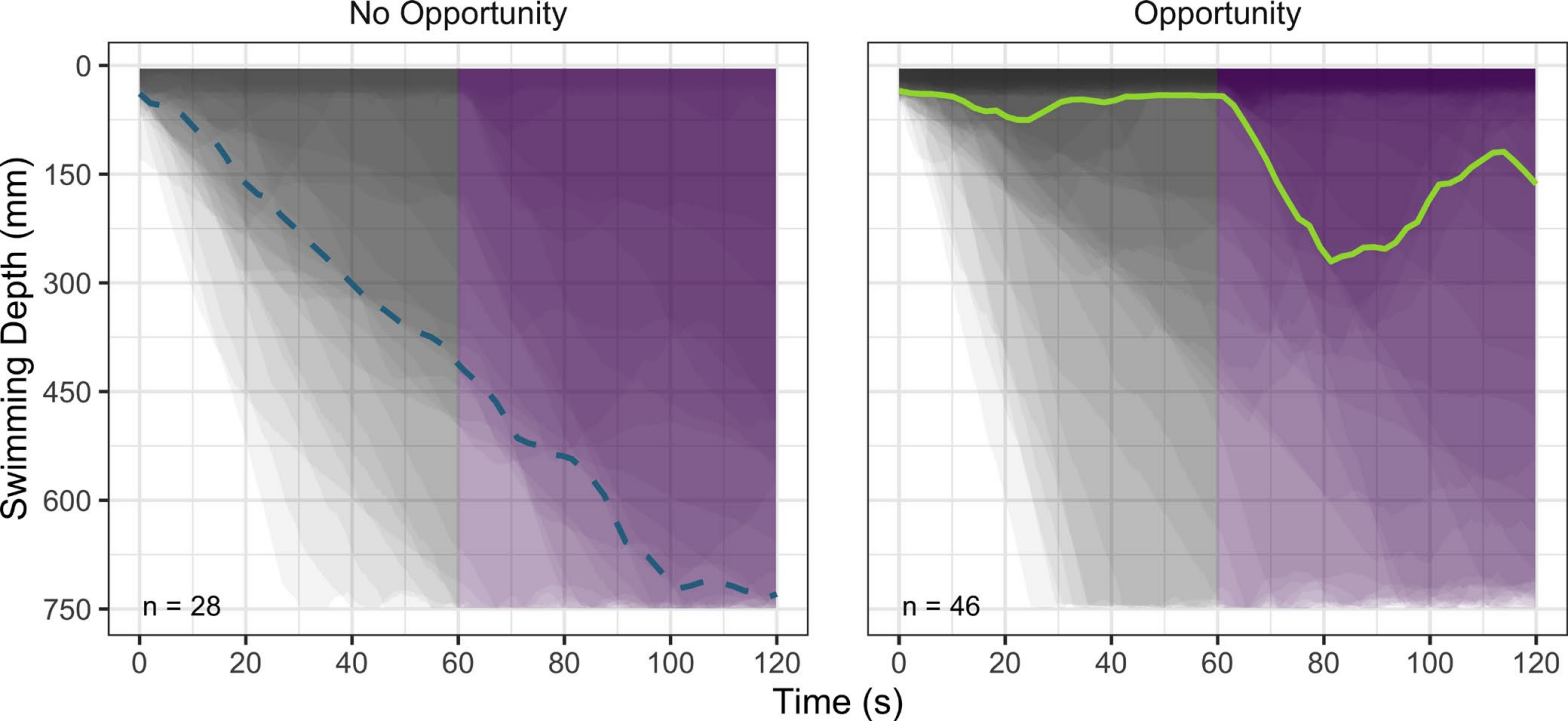
Individual swimming depths (areas plotted over one another) during experimental assays offering no feeding opportunity (left) and a food patch at the top (right). The median depths are indicated as colored lines, where the solid green line represents the median depth with an opportunity (food patch) and the dashed blue line shows the median without a surface feeding opportunity. The gray section (first 60 s) represents the acclimation phase, and the purple (last 60 s) denotes when individuals were exposed to a UVR threat from above

**FIGURE 2:**
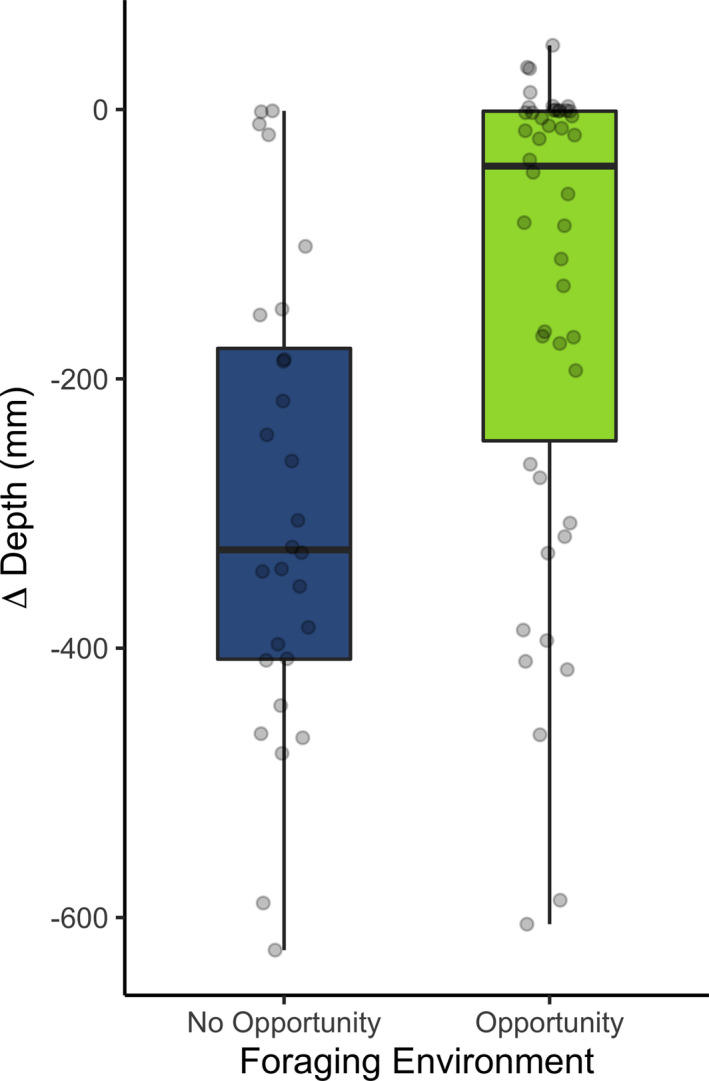
Change in median depth (∆ depth) from before (first 60 s) to during exposure to a UVR threat, showing that in the presence of food (opportunity) the response to UVR is weaker

When first entered into the recording arena, the *Daphnia* exposed to foraging opportunities remained higher in the water column than those without foraging opportunities (*U* = 846, *n*_[No Opportunity]_ = 28, *n*_[Opportunity]_ = 46, *p* = .024; Figure 1). Similarly, when exposed to a threat, in this case UVR exposure from the surface, more individuals stayed near the surface when offered a foraging opportunity (i.e., food patch) than when no such opportunity was present (*U* = 927, *n*_[No Opportunity]_ = 28, *n*_[Opportunity]_ = 46, *p* = .001; Figure 1). To ensure that differences recorded during UVR were not simply an artifact of autocorrelation with the acclimation phase, that is, the phase before the UVR threat, the change in depth between the two phases was also compared and the result persisted. That is to say, when offered a food patch *Daphnia* individuals were not responding as strongly to UVR as those without feeding opportunity at the surface (*U* = 318, *n*_[No Opportunity]_ = 28, *n*_[Opportunity]_ = 46, *p* < .001; Figure 2), meaning that animals swam slightly higher in the water column when the algae concentration was higher.

**FIGURE 3:**
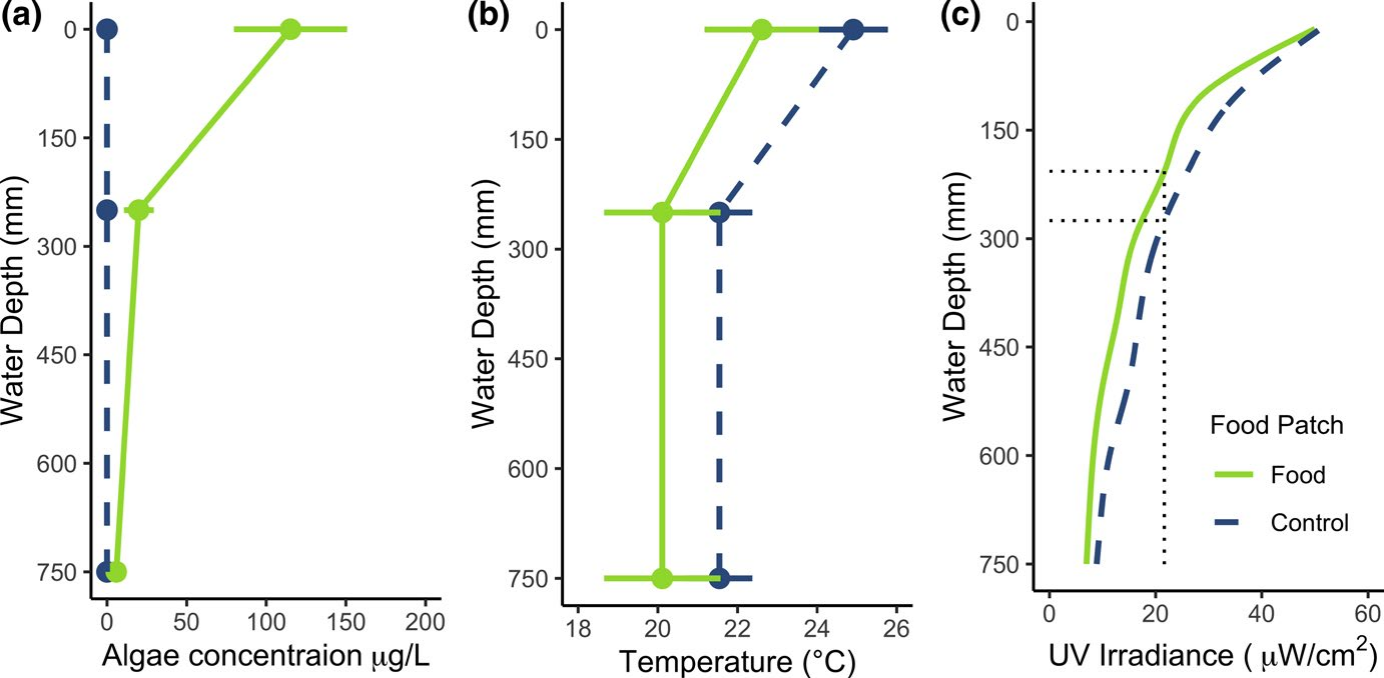
Physical characteristics of the experimental arenas. All figures have been rotated to most obviously display the spatial variation over depth of each parameter. (a) Displays algae concentration, (b) temperature, and (c) the ultraviolet irradiance. Each point represents mean ± 2 *SE* with green lines denoting the enclosure characteristics with foraging opportunities and the dashed blue lines representing the conditions with no foraging opportunities, that is, only a temperature patch. The dotted lines in panel (c) clarify that the difference is negligible between the two experimental conditions with regard to the depth of similar UVR threat; that is, the algal food patch did not affect the UVR attenuation through the water and thereby unlikely to have affected the behavior of the animals

Having established that *Daphnia* can sense the algal food patch at the surface and react accordingly, that is, show less avoidance to UVR in its presence, we calculated the mean of the average swimming depth for each recording block and checked the correlation of quantity of the algae at the surface of the water. We found that the mean swimming depth of *Daphnia* was marginally negatively correlated with the concentration of algae present in the food patch before (*F*_1,16_ = 4.193, *r* ^2^ = 0.208, *p* = .057) and during the UVR exposure (*F*_1,16_ = 4.494, *r* ^2^ = 0.219, *p* = .050). To ensure that the temperature gradient, introduced to create stratification, did not influence the swimming depth, we also checked the surface temperature of each experimental block and the mean swimming depth for that block and found no correlation either before (*F*_1,27_ = 0.002, *r* ^2^ = −0.037, *p* = .97) or during UVR exposure (*F*_1,27_ = 0.531, *r* ^2^ < 0.001, *p* = .47). Similarly, we tested whether the food patch affected the behavioral response through stronger UVR attenuation than without a food patch. Hence, at a depth with the same UVR irradiance (21.6 µW/cm^2^, which is the irradiance at the depth of mean chlorophyll; 207 mm; Figure 3), the frequency of individuals was negatively correlated with being above this depth without foraging opportunities, whereas it was instead positively associated with being above this depth in the presence of a foraging opportunity (*X* ^2^_[1,_ *_N_*_=74]_ = 4.095, *p* < .05).

## 4 DISCUSSION

Foraging is a critical feature among all animals, and how they trade‐off the opportunities of energetic gain versus the costs associated with risk is a central tenant of ecology (Brown & Kotler, 2004). Diel vertical migration, one of the largest foraging phenomena in terms of biomass, has long been studied in relation to predation, ultraviolet radiation (UVR) and even temperature (Lampert, 1989). To the best of our knowledge, there are no studies that explicitly, and at the individual level, test how the chronic stressor UVR interacts with spatial heterogeneity in opportunities for foraging. Therefore, the rationale of our study has been to advance our knowledge of individual zooplankton responses to this common and globally distributed threat, when also offered an opportunity to feed. Hence, our study mechanistically addresses how UVR and patchy food supply may affect the spatial and temporal variance in one of the largest daily biomass movements on Earth—diel vertical migration (DVM) of zooplankton. We fully appreciate that lab‐based studies are often far removed from natural conditions; however, we here demonstrate experimentally that a high‐density food patch is indeed a strong incentive for individual *Daphnia magna* to remain in surface waters, even in the face of the substantial risks UVR exposure imposes.

*Daphnia* are an integral member of most freshwater ecosystems worldwide, being a dominant grazer on phytoplankton and in turn, being a food source for higher trophic levels. They have a rapid output of offspring, taking approximately 10 days until maturity and then generating broods of parthenogenetically produced offspring every 3–4 days until death (Ebert, 2005). Hence, in accordance with our results and under the umbrella of the “pace of life syndrome” concept (Réale et al., 2010), *Daphnia* may perceive the risks associated with UVR exposure as less important than the acquisition of energy.

According to the transparency regulator hypothesis (TRH) (Tucker & Williamson, 2011; Williamson et al., 2011), differences in the physical environment such as UVR and algal abundances may modulate the depth that zooplankton are found at Williamson et al. (2011). In our experimental set‐up, the attenuation of UVR was negligibly affected by the food patch compared with the control environment. However, as a critical test that the reduction in UVR was not the driver of our findings, we checked the frequency of individuals above and below the UV irradiance equivalent to just below the food patch for both experimental conditions. We found a positive association between the food patch and individuals remaining at the surface and a stronger negative association with surface waters in the control conditions. This excludes the possibility that the food patch reduced the level of UVR, thereby providing refuge. Hence, we may conclude that UVR is less important in driving DVM when there are high densities of algae in surface waters despite the same sublethal UVR levels being prevalent. Similarly, previous studies have also indicated that temperature may affect the vertical distribution of zooplankton (Dawidowicz & Loose, 1992) and that they will, when not foraging, retreat to cooler waters and thereby reduce their metabolism. However, in our experiment we were unable to show that *Daphnia* distributed themselves to any thermal gradient, although we cannot discount the possibility of synergistic effects between temperature and algae.

Despite the clear statistical findings that the UVR, as well as the opportunity to feed in surface waters, affected the vertical distribution of *Daphnia*, we note that there is a high degree of inter‐individual variation within each experimental condition (see, e.g., Figure 1). This is very much in line with previous studies using the same methodology (Hansson et al., 2016; Heuschele et al., 2017), as well as the numerous reports showing the wide spatial distributions in natural ecosystems (Duffy, 2010; Stich & Lampert, 1981), suggesting that high variation in behavioral traits is a common and natural phenomenon under controlled conditions, as well as in the wild. There are multiple potential explanations for such commonly observed and high variances, and they are not necessarily mutually exclusive. One potential explanation is related to the asset protection principle (Clark, 1994), predicting that as the residual reproductive value (RRV) decreases (with age, for example), individuals will increase risk‐taking behaviors and, conversely, individuals with a high RRV will “play it safe” (Moschilla et al., 2018). Applying the RRV concept to our experiment may expose that the intraspecific variation in behavior is related to the energetic state of the individuals. In our study, however, we standardized the short‐term energetic state through creating competition‐free food availability, although we cannot exclude that age, reproductive state, or energetic reserves may have differed somewhat in our experiment, thereby adding to the variance.

Interestingly, we also noted a tendency for *Daphnia* to conform to one of two strategies. It appeared as though individuals either remained near the surface or resided near the bottom, with few individuals averaging at intermediate depths. Specifically, 75% of individuals without a foraging opportunity and 76% with a foraging opportunity occupied either the top or bottom 25% of the available space. This suggests that *Daphnia* may actually have “personalities” (or rather behavioral types) with some having a “bold” and others a “shy” attitude toward entering a novel environment (Heuschele et al., 2017), a phenomenon well‐known among mammals and other higher animals, for example, fish (Chapman et al., 2011).

In conclusion, our results suggest substantial intraspecific variation in threat response which, in a keystone species such as *Daphnia magna*, could have important consequences for communities and ecosystems (Duffy, 2010). Hence, in a broader context our results suggest that the UVR avoidance behavior, as well as the phenomenon of DVM, will be modified by *Daphnia* at the individual level depending on the food availability in surface waters, concluding that the DVM response to UVR will weaken and be traded off for food, a notion that may add to our understanding of the huge individual variance in DVM and avoidance behaviors in natural systems.

## CONFLICT OF INTEREST

The authors have no conflicts of interest to disclose.

## AUTHOR CONTRIBUTIONS

**Marcus Lee:** Conceptualization (equal); data curation (lead); formal analysis (lead); investigation (lead); methodology (equal); visualization (lead); writing – original draft (lead); writing – review & editing (equal). **Lars‐Anders Hansson:** Conceptualization (equal); formal analysis (supporting); funding acquisition (lead); investigation (supporting); methodology (equal); supervision (lead); writing – original draft (supporting); writing – review & editing (equal).

## Data Availability

The data are available in the dryad.org repository. https://doi.org/10.5061/dryad.41ns1rnd4.
